# Assessing drivers of implementing “Scaling-up the Systems Analysis and Improvement Approach” for Prevention of Mother-to-Child HIV Transmission in Mozambique (SAIA-SCALE) over implementation waves

**DOI:** 10.1186/s43058-023-00422-6

**Published:** 2023-07-24

**Authors:** Celso Inguane, Sarah Gimbel, Caroline Soi, Esperança Tavede, Filipe Murgorgo, Xavier Isidoro, Yaesh Sidat, Regina Nassiaca, Joana Coutinho, Maria Cruz, Mery Agostinho, Fernando Amaral, Aneth Dinis, Kristjana Ábsjörnsdóttir, Jonny Crocker, Nélia Manaca, Isaias Ramiro, James Pfeiffer, Maria de Fátima Cuembelo, Kenneth Sherr

**Affiliations:** 1 Department of Global Health, University of Washington, Seattle, WA USA; 2 Hans Rosling Center for Population Health, 700D.4, University of Washington, Box 351620, 3980 15Th Ave. NE, Seattle, WA 98105 USA; 3 Cooperativa de Ideias e Pesquisa em Saúde - CO-IDEAS, Maputo City, Mozambique; 4 Department of Child, Family and Population Health Nursing, University of Washington, Seattle, WA USA; 5 Manica Province Social Affairs Services, Chimoio, Mozambique; 6 Manica Province Health Directorate, Chimoio, Mozambique; 7 Comité Para a Saúde de Moçambique, Maputo City, Mozambique; 8 National Department of Public Health, Ministry of Health, Maputo City, Mozambique; 9 Center for Public Health Sciences, University of Iceland, Reykjavik, Iceland; 10 Department of Epidemiology, University of Washington, Seattle, WA USA; 11 Health Alliance International, Beira, Mozambique; 12 Department of Anthropology, University of Washington, Seattle, WA USA; 13 Department of Community Health, Faculty of Medicine, Universidade Eduardo Mondlane, Maputo City, Mozambique; 14 Department of Industrial & Systems Engineering, University of Washington, Seattle, WA USA

**Keywords:** Systems Analysis and Improvement Approach (SAIA), Scale-up, Mozambique, Consolidated Framework for Implementation Research (CFIR), Prevention of mother-to-child HIV transmission (PMTCT), Implementation science, Implementation determinants

## Abstract

**Background:**

The Systems Analysis and Improvement Approach (SAIA) is an evidence-based package of systems engineering tools originally designed to improve patient flow through the prevention of Mother-to-Child transmission of HIV (PMTCT) cascade. SAIA is a potentially scalable model for maximizing the benefits of universal antiretroviral therapy (ART) for mothers and their babies. SAIA-SCALE was a stepped wedge trial implemented in Manica Province, Mozambique, to evaluate SAIA’s effectiveness when led by district health managers, rather than by study nurses. We present the results of a qualitative assessment of implementation determinants of the SAIA-SCALE strategy during two intensive and one maintenance phases.

**Methods:**

We used an extended case study design that embedded the Consolidated Framework for Implementation Research (CFIR) to guide data collection, analysis, and interpretation. From March 2019 to April 2020, we conducted in-depth individual interviews (IDIs) and focus group discussions (FGDs) with district managers, health facility maternal and child health (MCH) managers, and frontline nurses at 21 health facilities and seven districts of Manica Province (Chimoio, Báruè, Gondola, Macate, Manica, Sussundenga, and Vanduzi).

**Results:**

We included 85 participants: 50 through IDIs and 35 from three FGDs. Most study participants were women (98%), frontline nurses (49.4%), and MCH health facility managers (32.5%). An identified facilitator of successful intervention implementation (regardless of intervention phase) was related to SAIA’s compatibility with organizational structures, processes, and priorities of Mozambique’s health system at the district and health facility levels. Identified barriers to successful implementation included (a) inadequate health facility and road infrastructure preventing mothers from accessing MCH/PMTCT services at study health facilities and preventing nurses from dedicating time to improving service provision, and (b) challenges in managing intervention funds.

**Conclusions:**

The SAIA-SCALE qualitative evaluation suggests that the scalability of SAIA for PMTCT is enhanced by its fit within organizational structures, processes, and priorities at the primary level of healthcare delivery and health system management in Mozambique. Barriers to implementation that impact the scalability of SAIA include district-level financial management capabilities and lack of infrastructure at the health facility level. SAIA cannot be successfully scaled up to adequately address PMTCT needs without leveraging central-level resources and priorities.

**Trial registration:**

ClinicalTrials.gov, NCT03425136. Registered on 02/06/2018.

**Supplementary Information:**

The online version contains supplementary material available at 10.1186/s43058-023-00422-6.

Contributions to the literature

- Our study supplements the few empirical studies that have used the Consolidated Framework for Implementation Research (CFIR) systematically and comprehensively to assess drivers of implementation of interventions in low- and middle-income countries.
- Our research echoes the findings that suggest that adequately implementing and scaling-up health interventions, such as those to prevent mother-to-child HIV transmission (PMTCT) in health systems with structural resource limitations, need to leverage central-level resources and priorities.
- Our study combined the strengths of a qualitative method-based analytical approach and an implementation framework to foster implementation science contributions to inform timely decision-making about intervention implementation and reduce the know-do gap.

## Background

The Systems Analysis and Improvement Approach (SAIA) is an evidence-based package of systems engineering tools [1] implemented at the health facility level in low- and middle-income countries (LMICs) with high HIV burdens like Mozambique, originally implemented to maximize the effectiveness of the provision of prevention of Mother-to-Child HIV transmission (PMTCT) services [2]. Mozambique has made important improvements, including increasing the proportion of pregnant women initiating PMTCT from 10% to over 90% from 2013 to 2015 [3]. However, quality gaps constrain adherence and viral suppression of HIV, leaving children at risk of acquiring HIV; those gaps include 58% of women in universal antiretroviral therapy for PMTCT (Option B+) lost to follow-up at 6 months postpartum and less than half of those retained in care being virally suppressed (< 1000 copies/mL) at their first postpartum visit in Central Mozambique [3]. SAIA is implemented through five steps that include (a) understanding systems inefficiencies through cascade analysis, such as the PMTCT cascade analysis tool (PCAT) [1, 4], (b) guiding the identification and prioritization of low-cost workflow modifications through process mapping and workflow observation to identify bottlenecks and guide discussion on opportunities to modify workflow across the cascade, and (c) iteratively testing and redesigning those modifications through continuous quality improvement [2]. In a previous study across three African countries (Côte d’Ivoire, Kenya, and Mozambique), SAIA significantly increased maternal antiretroviral initiation and early infant screening of HIV [5] and was found to be a scalable model with the potential to maximize benefits of universal antiretroviral treatment for women and their babies [5]. SAIA has been adapted to improve the continuum of care for other cascades, such as adolescent and pediatric HIV, family planning, hypertension, and mental health [6–12].

Determinants of intervention implementation identified across PMTCT and adult HIV cascades in Sub-Saharan Africa highlight the need to attend to the influence of intervention stakeholders, the resource needs of intervention beneficiaries and local health systems, and how intervention are implemented. For instance, determinants of successful implementation of PMTCT interventions were related to networks and communication which facilitated intervention implementation in high-performing facilities through clear communication of roles across sectors [13] but also through health workers communicating and contacting with patients [14]. Available resources, especially stockouts of laboratory resources and testing supplies [15]; available physical space; and human resources were barriers to implementing PMTCT and other HIV-related interventions in low-performing facilities in three Sub-Saharan African countries [13]. Healthcare workers’ self-efficacy was also relevant in influencing implementation in other PMTCT interventions across Sub-Saharan Africa, through finding solutions to overcome implementation challenges [13–15]. Three determinants were related to the implementation process, including external change agents. In Mozambique, the study team was not perceived as influencing engagement but was associated with performance in Kenya and Côte d’Ivoire [13]. The facility staff in Kenya did not mention if SAIA was implemented as planned, but in the other two countries, the staff reported implementation as planned in high-performing facilities [13]. Reflecting and evaluating expressed by establishing formal and informal feedback and accountability mechanisms [15] which SAIA encouraged through updating flow maps and PCAT, and through iterative continuous quality improvement, although it was limited by a shortage of staff at weaker health facilities [13]. Barriers include concerns about privacy and stigma and the limitations of the healthcare system including healthcare worker attitudes [14].

As for intervention implementation, the scale-up of HIV-related interventions across high-income and LMICs calls for the need to attend to the importance of contextual factors that mediate intervention uptake and scale-up [16–18]. A comparative analysis of the scale-up on an mHealth intervention that uses SMS communication to improve patient adherence to medication and engagement in care in Canada and Kenya showed that, even with robust research evidence, scale-up was a precarious and uncertain process, embedded within the wider politics and financing of Canadian and Kenyan health systems [16]. Key factors that contributed to the successful scale-up of a treatment as a prevention intervention include stakeholder buy-in, including government symbolic investments on the intervention grounded on perceptions about quality and strength of evidence supporting the intervention [17], which highlights the importance of packaging interventions for greater impact and relevance [16]. Additional determinants, identified through differentiated service delivery models across the HIV continuum of care and treatment, include focusing on investing resources that can address patient differentiated needs, ensure sustainable inclusion of more health worker categories (especially community health workers), and strengthen supply chains [18]. The personal attributes of intervention leaders and implementers are important determinants. This includes highly motivated and social justice-oriented individuals who facilitate the implementation of evidence-informed adaptations across a highly decentralized service delivery system using program data [17].

Building on the original SAIA findings, SAIA-SCALE was designed to evaluate a novel dissemination approach where the intervention is delivered by district health system managers (rather than research nurses) as a foundation for further scale-up. Study procedures have been previously published [3]. The SAIA-SCALE mixed methods evaluation was guided by the Reach, Effectiveness, Adoption, Implementation, and Maintenance (RE-AIM) model [19]. To identify salient implementation determinants, the Consolidated Framework for Implementation Research (CFIR) [20] was embedded in the qualitative evaluation. As a meta-theoretical determinants framework, the CFIR assumes that the five domains of intervention characteristics, outer setting, inner setting, characteristics of the individual, and implementation process interact to influence implementation processes and outcomes [20, 21]. The suggested addition of a sixth domain, “characteristics of systems,” and of six constructs associated with that new domain adds to efforts to improve CFIR’s contributions to implementation science’s capacity to aptly describe implementation drivers and helps expand the framework’s applicability to LMICs [22], including Mozambique [13, 23–26]. Yet, CFIR’s potential contribution to implementation science’s efforts to describe implementation determinants is limited in that the majority of empirical studies using CFIR are applied post-implementation and used to guide data analysis, rather than more holistically, and by only some of that literature providing the rationale for selecting and using CFIR constructs and subconstructs [21, 22].

This manuscript reports on determinants (facilitators and barriers) of SAIA-SCALE’s implementation after 12 and 24 months of implementation applying the CFIR (1) during the implementation of the intervention and (2) for multiple purposes including to guide data collection, analysis, and interpretation. We used CFIR systematically by providing our rationale for selecting specific constructs and subconstructs, and by highlighting those constructs with influence on the successful implementation of SAIA.

## Methods

### Study design

We used an extended case study approach that combined (1) a case-oriented approach that provides health facility-specific findings that could guide decision-making at the health facility level, with (2) a variable-oriented approach which assessed similarities and connections across intervention health facilities [27–31]. We embedded conceptual entities from the CFIR (domains, constructs, and subconstructs) within the extended case study design to inform the development of data collection tools and analysis.

### Intervention design

Briefly, SAIA-SCALE was a stepped wedge cluster randomized trial implemented in Manica Province, Central Mozambique, from April 2018 to March 2021. SAIA-SCALE covered 12 districts and three health facilities per district (a total of 36 facilities), with phased in implementation across three waves of 12 months each [3]. Four districts were randomly allocated to the intervention wave. For the first 12 months of implementation in each wave (the intensive phase), research nurses accompanied district managers to support SAIA cycles in subordinate facilities, followed by a maintenance phase whereby district managers received only financial support to lead SAIA cycles. Modest flexible funding was provided to district managers to support facilities to implement quality improvement plans. Before initiating the intensive phase, district maternal and child health (MCH) and PMTCT managers attended a week-long training on the SAIA implementation strategy, which includes an introduction to SAIA analysis and improvement tools, implementation schedule; and data collection procedures [3]. This enables managers to lead 2-day-long trainings on SAIA to frontline PMTCT staff and facility managers [3]. Within each district, SAIA is introduced one facility at a time, until all three facilities are covered in each district. In the first 2 months after the introduction of the intervention, district managers visit implementing health facilities every 2 weeks, after which a 10-month-long intensive implementation phase and a 2-year-long maintenance phase follow [3]. Throughout the first year of implementation, study nurses accompany district supervisors during health facility visits. Analysis and improvement cycles are conducted monthly, with an average of 12 cycles a year per facility [3]. Study nurses and district supervisors visit to health and other activities related to the intervention at health facilities are funded through a small grant of approximately $1500 per facility per annum [3].

### Study setting and characteristics of participants

Study participants were purposively selected from 21 primary healthcare facilities out of seven districts of Manica Province (Chimoio, Báruè, Gondola, Macate, Manica, Sussundenga, and Vanduzi), over two rounds of data collection. Two district capitals are (urban) municipalities (Chimoio and Gondola), and four health facilities are urban, while 17 are rural. They are all primary healthcare facilities, led by doctors and supervised by district health services. Manica Province is located in central Mozambique and has a population of approximately two million, 12 districts and 118 health facilities which cover over 98% of the healthcare needs of the population [3]. All health facilities provide primary healthcare, HIV testing in antenatal care, and antiretroviral therapy for PMTCT, and Option B+ only covers 60% of facilities in the province [3]. Mozambique’s national health system is organized into four levels of healthcare attention (primary, secondary, tertiary, and quaternary) with corresponding geographic levels of management (rural or urban, city or district, province, and regional). The district, which in some cases is also a municipality, is the most basic level of administrative and financial management of Mozambique’s health system, instead of the health facility. While PMTCT is part of HIV, one evidence of its priority is that is managed through its own program, separate from the national HIV program.

The first round (April 2019) covered a 12-month wave of implementation in four intensive phase districts and 12 health facilities (three per district), from April 2018 through March 2019. The second round (March 2020) covered a 12-month wave of implementation in three newly activated intensive phase districts and nine health facilities, as well as the four districts and 12 health facilities that had been in the intensive phase in the previous 12 months that transitioned to the maintenance phase in months 13–24. This second wave comprised intervention implementation from April 2019 to March 2020. Two district-level health managers from each district were eligible (the district MCH supervisor and the PMTCT focal point), and frontline nurses and facility MCH managers were eligible from each study facility. Study eligibility included working as a government employee involved in PMTCT delivery or its oversight for at least 12 months preceding data collection. Interviewers approached each potential participant to assess eligibility and eligibility before each interview and FGD and included participants who were available at the time of the interview or FGD.

We included 92% (*n* = 85/92) expected participants from 21 health facilities and seven districts: 50 participants through face-to-face IDIs and 35 through three face-to-face FGDs (Table 1). Nearly all participants were women (98%), mostly frontline nurses (49%) and health facility MCH managers (33%). No potential participant declined to participate; however, seven potential participants were not included for lack of eligibility, including being new in their position, lacking knowledge about the intervention, not being a government employee, and having been interviewed in another participant category. The number of interviews we conducted is enough to ensure saturation on the main themes (code saturation) at the health facility manager level and to ensure saturation at the more detailed thematic levels (sub-themes) at the district managers and frontline nurses levels (meaning saturation), given that code saturation at 80–90% estimates range from 8 to 16 interviewees, while meaning saturation ranges from 16–24+ interviews [32–34].

**Table 1:** Study participants in SAIA-SCALE, Mozambique 2018–2020

**Participants**	**IDI**	**FGD**	**Total** **: ** ***n*** ** (%)**	**Women** **: ** ***n*** ** (%)**
District managers	17	0	17 (20.5)	17 (100.0)
MCH facility managers	11	16	27 (32.5)	26 (96.3)
Frontline nurses	22	19	41 (49.4)	40 (97.6)
**Total**	**50**	**35**	**85 (100.0)**	**83 (97.7)**

### Data collection

Two teams of three qualified interviewers, supervised by two study investigators, led the data collection over 2 weeks in 2019 and 3 weeks in 2020. Interviewers had at least a bachelor’s with an honors degree in social sciences, humanities, or public health and at least 3 years of research experience in maternal and child health or HIV. Data collection was conducted at health facilities, district health offices, and the implementing NGO offices, following 5 days of training on human subjects, data collection and data management procedures, and pre-testing of data collection and supervision instruments.

Team composition, supervision, and debriefing meetings were used to improve data quality and analysis. One-hour-long IDIs and FGDs were conducted in Portuguese. For each IDI, one team member conducted the interview while a second member documented it through field notes and audio recording (if participants consented; in cases where consent was not provided for audio recording field notes were maintained by the second team member). FGDs were run by all four team members: one facilitator, two note-takers, and an observer (study investigator). Study investigators observed at least one interview led by each interviewer and provided feedback on the quality of rapport and interview techniques, time spent during the interview, and how participant anonymity and data confidentiality were ensured. The study investigator used ethnographic debriefing techniques [35] to help teams supplement collected data or elicit data they had collected but that had not been documented in field notes or audio recordings.

### Data analysis

At the end of each data collection period (April 2019 and May 2020), the study team obtained feedback from district managers and HIV staff on preliminary findings synthesized based on field notes and audio recordings. An in-depth analysis was carried out in January through February of 2020 and 2021 when transcribed audio recordings were available. Data analysis followed a consensus-based and iterative approach (deductive and inductive) based on a conceptual framework grounded on the CFIR (Table 2). Data was translated from Portuguese into English immediately before data analysis. We presented preliminary findings to stakeholders in reports written in Portuguese, the official language of Mozambique. Two study investigators translated those reports and data segments that we included in the analysis memos that we used to develop this manuscript.

**Table 2:** Conceptual framework used for SAIA-SCALE data analysis, across two intervention waves in Mozambique, 2018–2020

**CFIR domains**	**Constructs and subconstructs**
**Intervention characteristics**	Intervention source
	Relative advantage
	Adaptability
**Outer setting**	Peer pressure
	External policy and incentives
	Needs and resources of those served^b^
**Inner setting**	Structural characteristics
	Available resources^b^
	Networks and communications
	Compatibility (subconstruct of implementation climate)
	Relative priority (subconstruct of implementation climate)
	Access to knowledge and information (subconstruct of readiness for implementation)
	Leadership engagement (subconstruct of readiness for implementation)^b^
**Characteristics of individuals**	Knowledge and beliefs about the intervention
	Self-efficacy
**Process**	Planning
	Innovation participants (subconstruct of engaging)
	Executing
	Linkages among intervention components^a^

*CFIR* - Consolidated Framework for Implementation Research

^a^Construct is not original to CFIR but is based on the investigators’ experience using CFIR

^b^Conceptual entities added during data analysis, not initially prioritized by study investigators

As part of the deductive approach, we guided our analysis by a codebook containing a preselected list of 16 constructs and subconstructs organized around the five original CFIR domains of intervention characteristics, outer setting, inner setting, characteristics of individuals, and [implementation] process (https://cfirguide.org/tools/tools-and-templates/). Study investigators had prioritized those constructs and subconstructs based on their research experience using the CFIR in the intervention’s geographic area. The 16 constructs included the “linkages among intervention components” construct, which was not originally in the CFIR but was based on the investigators’ experience using the framework. We added a construct (available resources) and a subconstruct (leadership engagement) that we had not anticipated, but that emerged during data analysis (inductive approach), because they reflected themes that emerged from study participants during IDIs and FGDs.

We conducted data analysis with the aid of ATLAS.ti, versions 8.4 and 9 (Scientific Software Development GmbH, Berlin, Germany), where we stored data and the codebook, including definitions of conceptual entities. To ensure consistency in the analysis, two investigators piloted the analysis procedures in 2-day-long workshops, after which they independently analyzed the same data (IDI and FGD transcripts and notes and field notes) for each study facility. They met weekly to resolve discrepancies in coding if those emerged and produced joint case memos per facility and a joint report for each implementation wave. Instead of using intercoder reliability, study investigators used a consensus and tiebreaker-based system that was effective in previous analysis projects [36]. Specifically, whenever consensus was not reached about coding data segments, a third investigator with experience conducting analysis using the CFIR resolved coding disagreements. Then, one of the two investigators conducting the analysis entered the agreed-upon codes and ratings into ATLAS.ti, after which the two investigators met to write (intensive and maintenance) phase and wave memos.

Ratings followed an approach developed by Damschroder et al., which defines the valence and strength of each CFIR construct or subconstruct [20, 37]. Valence denotes the positive or negative influence of the construct or subconstruct on implementation [20, 37], which we defined as a facilitator or a barrier. Strength indicates (1) the level of emphasis, which is determined by the descriptive language participants used; (2) whether concrete examples were provided; and (3) the level of participant agreement on language and/or examples [20, 37]. Positive valence is indicated by +, and its strength can be weak (+1) or strong (+2), whereas negative valence is indicated by −, and its strength can be weak (−1) or strong (−2) [20]. The valence of constructs and subconstructs can also be neutral (0) if they have unclear directional influence, and their influence can be mixed (X) if the positive and negative influences cancel each other out [20, 37]. A construct or subconstruct was deemed significant in an intervention phase if at least 2 participants mentioned it and was considered important to an intervention wave if it was mentioned in both intervention phases within the wave.

### Ethical considerations

The study was approved by the Institutional Review Boards (IRBs) of the University of Washington and Mozambique’s Eduardo Mondlane University Medical School, as well as Mozambique's Ministry of Health. IDIs and FGDs were conducted after obtaining written informed consent from study participants. Participants were not given incentives; however, FGD participants received refreshments, as is consistent with research practice in Mozambique. We protected audio recordings and field notes using individual alphanumeric codes that prevented the positive identification of each study participant. Before preparing this manuscript, we obtained stakeholder feedback on preliminary study findings and incorporated that feedback into the current manuscript. We maintained human subjects’ protection procedures, with emphasis on preserving participant anonymity and data confidentiality, at all stages of the study, from data collection through data analysis and reporting. For this reason, in this manuscript, we do not connect findings to individual health facilities or districts. Therefore, although we report on this study using the Consolidated Criteria for Reporting Qualitative Research (COREQ), which is appropriate when collecting data through IDIs and FGDs [38], as we did, we could not fill some of the sections of the COREQ checklist (Additional file).

## Results

### Facilitators of successful implementation

The main facilitator of the successful implementation of the intervention, regardless of intensive or maintenance phase, was compatibility of the intervention with organizational structures, processes, and priorities of Mozambique’s health system at the district and health facility levels (Table 3).

**Table 3:** Facilitators of successful implementation of SAIA-SCALE, across two intervention waves in Mozambique, 2018–2020

**Facilitators in both implementation waves (2018–2019 and 2019–2020)**	
** Inner setting domain**	
Compatibility (subconstruct of implementation climate)	SAIA compatibility with organizational structures, processes, and priorities of national health service at district and facility levels
**Facilitators in the second implementation wave (2019–2020)**	
** Intervention characteristics domain**	
Relative advantage construct	Relative advantage to MCH and PMTCT cascade at the health facility
** Outer setting domain**	
External policy and incentives construct	Support and pressure that provincial managers exerted over district and facility managers and personnel
** Inner setting domain**	
Relative priority (subconstruct of Implementation climate)	Relative priority of intervention at district and facility levels, supported by PMTCT being a national priority in Mozambique
Leadership engagement (subconstruct of readiness for implementation)	District managers’ active involvement in intervention activities: health facility supervision, technical support, and solving logistical issues
** Implementation process domain**	
Executing construct	Implementation of intervention as originally planned, with high-quality methods and committed personnel

Some participants reported that the intervention helped implement or improve the implementation of already planned activities at the health facility and did not conflict with activities underway at district and health facility levels. This made frontline nurses and managers look at the intervention as part of their “routine and a necessary activity—this is our daily bread” [Nurse manager, March 2020]. Other interviewees described that the intervention was a good fit because—by consulting with community leaders—it respected the community engagement principle that is central to Mozambique’s health system. Participants echoed these thoughts as follows:

The intervention is compatible with how the health facility works because it doesn’t interfere; it helps us improve and see our weaknesses. For instance, in the maternity ward we were not able to meet our target of bringing mothers to the maternity. They [intervention leaders] gave us tips and we are meeting our targets - Frontline Nurse, Urban Health Facility, April 2019.

The intervention is compatible because nurses don’t feel that it is something that was added to their work; they view SAIA as a normal activity - Frontline nurse, Rural Health Facility, March 2019.

Facilitators of successful implementation of the intervention that were limited to a year after implementation (intensive phase alone) were related to the relative advantage of the intervention, external policy and incentives, the relative priority of the intervention, and the quality with which the intervention was implemented.

Study participants mentioned that the intervention’s advantages to the MCH program and to the PMTCT continuum of care at health facilities, including improving MCH managers and frontline nurse awareness of the need to conduct data quality assessments, to improve MCH data quality and PMTCT indicators such as early enrollment of mothers into antenatal care, early collection of blood samples for early infant diagnosis testing, and retention of mothers and their children in the PMTCT continuum of care. They also noted that the intervention improved frontline nurse and district manager competencies for independently identifying and solving problems in a timely fashion, which also promoted nurse performance and self-evaluations. Finally, participants added that the small grant funding provided to the districts helped improve the quality of MCH services at health facilities because it helped purchase medical equipment and furniture, patient clothing, and other items. The following statement summarizes some intervention advantages in the second intervention wave.

[SAIA-SCALE] brought many advantages, because before due to the lack of funding we were unable to go to health facilities in the periphery. We receive funds through the sub agreement to purchase office and hygiene supplies and supplies to help prevent infections. So, with SAIA-SCALE we only have advantages. Before SAIA-SCALE we had nurses who didn’t know the advantage of doing [HIV] prophylaxis to a child and the advantage of correct follow up for antiretroviral therapy during pregnancy and breastfeeding. Therefore, with this study that we conduct every moth to our data, even nurses who didn’t do data checks every month can now do it. We purchase bed linen, which was a need. now we have bed linen to prevent infections, we have hygiene and cleaning supplies that also help us prevent infections. SAIA-SCALE brought many things for us – District manager, March 2020.

They summarized that the intervention helped improve the humanization of services, because it inspired frontline nurses and managers to focus more on the health of mothers and their HIV-exposed babies. Expression of the role of external policies and incentives as a facilitator were support and pressure that provincial managers exerted over district and health facility managers and other personnel. Study participants noted that both study nurses and provincial MCH and PMTCT managers provided technical support and were rigorous in demanding professional excellence from frontline nurses and MCH nurse managers.

The project is being implemented by people with knowledge, competencies and who use adequate methods – [study] nurses and district managers. The methods they use [include] respect, demand [for excellence], patience, and tips so we can improve our work. This method is different from the method used by [another NGO] workers, who are solely concerned with whether there is data, they demand from us and shout at us, forgetting that nurse’s salary is paid for by the State [government], not by [the NGO] - Frontline nurse, Rural Health Facility, March 2019.

They highlighted that while study nurses provided technical support whenever health facility nurses and managers asked for it, they had a participatory leadership style that helped frontline nurses improve their own leadership skills. Specifically, they noted that study nurses always gave health facility nurses the opportunity to chair monthly health facility meetings. They added that the provincial MCH and PMTCT managers conduct weekly monitoring of PMTCT indicators, thanks to data they receive through the provincial MCH WhatsApp group. The provincial MCH manager supplements weekly monitoring with surprise visits to health facilities, during which she always communicated her presence to the district manager when she arrives at the district.

The organizational (or inner) setting had a positive influence on successful implementation because stakeholders at the health facility and district level regarded the intervention as a priority, and intervention leaders and other managers at the health facility and district level were actively involved. That the intervention addresses PMTCT, a national priority in Mozambique, might influence it to be regarded as a priority at lower levels of the country’s health system. For instance, community members were engaged in community mobilization through health committees and joint management committees that gave biweekly talks at the community on various topics, including on the need for male partner involvement in MCH consultations at the facility which, given gender power relations could contribute to increasing women’s access to MCH services. Additionally, study participants noted that all MCH personnel at the facility and district levels continued to be involved in the intervention, including nurses, managers, clinicians, and health facility managers. Study participants noted, however, that antenatal care managers seemed to be more active, perhaps because that section is the point of entry for mothers and babies at the MCH program. District managers also continued to be involved in the intervention, by conducting health facility supervisions, providing technical support, and finding solutions for logistical challenges that could affect intervention implementation, such as facilitating access to transportation for supervision visits. Study participants found the involvement of chief medical officers particularly important because that had not been planned for in the intervention. However, in some districts, the intervention seemed to be less of a priority for part of the administrative personnel, whom some participants noted did not always ensure timely disbursement or use of the small grant funds provided to districts. Study respondents noted that program personnel (e.g., district MCH supervisors) had limited influence on administrative decisions at the district level (including the use of the small grant funds).

Finally, the implementation process positively influenced the implementation of the intervention through staff involved in program execution as planned in the protocol. Study participants noted that the intervention used appropriate methods and was implemented by highly qualified and committed personnel (PMTCT and MCH district supervisors and study nurses) who displayed an exemplary work ethic. They noted that all district managers were competent in using intervention tools, such as the cascade analysis tool, and they coupled solidarity and respect for health facility managers and nurses with demands for professional excellence. An instance of solidarity that frontline nurses mentioned was that whenever study nurses and district managers visited health facilities for the monthly meeting, before conducting intervention-specific activities, they helped health facility staff find solutions for problems identified through the facility action plan, and helped nurses manage the patient flow.

We go to the health facility very early [in the morning]. We provide technical support, [which includes] working to try and reduce [patient] flow, so we can manage to have her [nurse] participate in the meeting. So, after our work, we go ahead, we register, we collect the monthly data. We register monthly data in the giant paper. Only they [nurses] come. We register data in the PCAT when all of them are in the meeting and we write the work plan in that paper – District manager, March 11, 2019.

### Barriers to successful implementation

Barriers to intervention implementation regardless of intervention wave and phase were related to the lack of adequate health facility infrastructure and resources to satisfy the needs of patients who seek MCH or PMTCT services at health facilities and to challenges that some districts had in managing the small grants provided by the intervention (Table 4).

**Table 4:** Barriers to the successful implementation of SAIA-SCALE, across two intervention waves in Mozambique, 2018–2020

**Barriers in both implementation waves (2018–2019 and 2019–2020)**	
** Outer setting domain**	
Needs and resources of those served	HIV-positive mothers do not show up for institutional births because of inadequate health facility and road infrastructure
** Inner setting domain**	
Compatibility (subconstruct of implementation climate)	Challenges that some districts had in managing the small grants provided by the intervention
Available resources (subconstruct of readiness for implementation)	Inadequate health facility infrastructure: small rooms, lack of privacy, lack of registries and medical and hygiene suppliers and child delivery medical equipment
	Some health facilities did not have running water and health workers interrupted work to fetch water
**Barriers in the second implementation wave (2019–2020)**	
** Inner setting domain**	
Available resources (subconstruct of readiness for implementation)	Stockouts of medicines for HIV prophylaxis and ART at facility and district prevent meeting PMTCT targets

The lack of adequate health facility and road infrastructure, and the distance between health facilities that reduce HIV-positive mothers’ access to MCH and PMTCT services at the health facility level, was identified as an important barrier. Specifically, the small size and lack of quality of pregnant mothers’ waiting homes [*casa de espera da mãe grávida*] and the lack of privacy during MCH consultations discourage mothers from seeking institutional births. This was compounded by the distance between the residences of those mothers and the health facility and the poor road quality. Some health facilities lacked running water, and frontline nurses often interrupted their work to fetch water, which distracted healthcare workers from focusing on improving the quality of data and of healthcare service provision.

Additionally, the distance from health facilities to residential areas and lack of transportation support from the district health authorities prevented health workers from conducting community outreach work that study participants believe could contribute to increasing mothers’ access to MCH and PMTCT services. Study participants also mentioned that some districts had challenges in managing and disbursing funds from the small grants provided by the study:

It takes about 3 months to have those funds released [intervention small grants]. What we would like to ask in the management of those materials is that when they go and purchase them, the person responsible for maternity wards at the district level should be part of the team that purchases the material along with the administrative person, so we can have materials with quality. Because usually you ask for a basin and they bring you a basin to wash hands when you need a basin for the delivery. So, this is not helpful. […]. You ask for a trash bin and they bring you a backet That doesn’t make any sense. They shouldn’t include the nurse only when they go shopping. They should also include her when they do [procurement] to identify which items have quality. [Otherwise, the] administrative person can think in his/her head that the basins he/she has chosen are the same. This is what happens when materials and supplies are purchased – FGD with maintenance phase nurses from four districts, March 14, 2020.

The only barrier to successfully implementing the intervention after a year of implementation was related to constant stockouts in the ART combination of nevirapine and azidothymidine (AZT) that is used for prophylaxis and treatment of HIV in babies that were being assisted in the PMCT continuum of care. Study participants noted that these stockouts could make prophylaxis ineffective and compromise the prevention of vertical transmission of HIV, one of the main intervention targets. Study participants noted that these stockouts happened at both health facility pharmacies and at the district depots.

## Discussion

We used the CFIR to identify determinants (facilitators and barriers) of scaling up the Systems Analysis and Improvement Approach for the Prevention of Mother-to-Child Transmission of HIV (SAIA-SCALE) [2], across two implementation waves that included an intensive phase (2018–2018) and an intensive and maintenance phase (2019–2020) in Mozambique, a low-income country with one of the highest burdens of HIV globally. We shared preliminary findings with stakeholders from the seven districts included in the study, to facilitate timely decision-making about intervention implementation, including adjusting the implementation approach. These findings can also serve as a reference for assessments of subsequent SAIA-SCALE implementation phases.

The relative advantage of the intervention and the role of external policy and incentives, the relative priority of the intervention, and the quality with which the intervention was implemented only facilitated intervention implementation in the second implementation wave (April 2019 through March 2020) but did not facilitate in the first implementation wave which only included intensive phase health facilities. Other studies that have used the CFIR systematically in other LMICs, as we did, have also identified relative priority as a facilitator of intervention implementation [23, 39]. Conversely, we identified compatibility with organizational structures, processes, and priorities at the primary healthcare and health system management level as a facilitator, but other studies have identified it as a barrier [39]. While we identified the involvement of community actors as an important mediator of the compatibility of the intervention with the local health system, other studies that applied the CFIR in Mozambique could not ascertain the influence of community involvement in implementation [23]. However, studies conducted across Sub-Saharan Africa have shown that the mediating role of community actors is facilitated by community health workers [14].

Barriers to successfully implementing SAIA-SCALE regardless of intervention wave and phase were more widespread and were related to the lack of adequate health facility and district infrastructure and resources to address the needs of patients who seek MCH or PMTCT services. They were also related to challenges that some districts had in managing the small grants provided by the intervention. Our identification of available resources as a barrier to successfully implementing interventions, especially related to inadequate resources in the health systems of LMICs has also been highlighted in other LMICs [39]. It is also consistent with the original SAIA trial which found that “investments in infrastructure and human resources [could] be critical to improve prevention of mother-to-child HIV transmission service delivery and protect infants from HIV” [40], particularly in low-performing health facilities in other African countries, including where SAIA-SCALE was implemented in Mozambique [13]. These findings are also consistent with those documented in other studies on the scale-up of pediatric and adult HIV interventions across the world, which highlight the shortcomings of believing in the technological fix and call for the need to attend to the importance of contextual factors that mediate intervention uptake and scale-up [16–18].

Our study supplements the relatively small proportion of empirical studies that have used CFIR systematically and comprehensively. We applied the CFIR in annual qualitative data collection throughout implementation, whereas most studies have used it after implementation [21, 22]. Similar to other studies conducted in LMICs [22], we used the CFIR for multiple purposes, including to guide data collection, analysis, and interpretation of the findings. To facilitate intervention stakeholders to use findings to inform intervention adjustments if needed, by health facility or district and across districts, we used an extended case study design. This study design has been successfully used across the social sciences and the humanities, including in healthcare settings, to understand the unique characteristics of social settings, and commonalities and differences across those settings [27–30]. In doing so, we combined the strengths of qualitative method-based analytical approaches and an implementation framework to foster implementation science contributions to inform timely decision-making about intervention implementation.

### Limitations

The likelihood of social desirability bias that is common when obtaining self-reported data was reduced by triangulating data collection methods (i.e., individual interviews and FGDs) and by using a robust analytical approach that included sharing findings with stakeholders and combining consensus-based analysis with a tiebreaker system. Our findings and discussion cannot cover the full scope of recent extensions to the CFIR, which were made after we had developed the study protocol [22]. We took this decision to preserve analytical consistency, by focusing on the same constructs and subconstructs across the study stages of data collection, analysis, and interpretation.

## Conclusions

Using qualitative methods, we conducted an evaluation of the implementation of SAIA-SCALE in Mozambique that suggests that SAIA’s scalability for PMTCT depends on the intervention’s amenability to fit within organizational and management structures and processes, and with the priority given to PMTCT at the health facility and district. The availability of adequate health facility and district infrastructure to cater to the needs of MCH and PMTCT service users was also important and falls beyond the capacities of the primary level of healthcare provision and health systems management in Mozambique (it is the purview of the central level). This implies that, ultimately, SAIA or other PMTCT interventions cannot be successfully scaled up to adequately address PMTCT needs without leveraging central-level resources and priorities that focus on strengthening the health system.

## Supplementary Information

**Additional file 1.** COREQ (COnsolidated criteria for REporting Qualitative research) Checklist.

## Data Availability

The data generated during this study are not publicly available for ethical reasons related to protecting participant confidentiality, as stated in the consent forms approved by the IRBs that approved this study. However, the corresponding author can provide those data upon reasonable request, if needed.

## Abbreviations

CFIR: Consolidated Framework for Implementation Research
PMTCT: Prevent of Mother-to-Child HIV transmission
SAIA: Systems Analysis and Improvement Approach
SAIA-SCALE: Scaling-up the Systems Analysis and Improvement Approach’ for Prevention of Mother-to-Child HIV Transmission in Mozambique
LMIC: Low- and middle-income country
MCH: Maternal and child health
RE-AIM: Reach, Effectiveness, Adoption, Implementation, and Maintenance
IDI: In-depth individual interview
FGD: Focus group discussion
IRB: Institutional Review Board
NGO: Non-governmental organization

## References

[1] Wagner AD, Crocker J, Liu S, Cherutich P, Gimbel S, Fernandes Q (2019). Making smarter decisions faster: systems engineering to improve the global public health response to HIV. Curr HIV/AIDS Rep.

[2] With input from the SAIA Study Team, Sherr K, Gimbel S, Rustagi A, Nduati R, Cuembelo F, et al. Systems analysis and improvement to optimize pMTCT (SAIA): a cluster randomized trial. Implement Sci. 2014;9(1). Available from: http://implementationscience.biomedcentral.com/articles/10.1186/1748-5908-9-55. Cited 2020 Feb 20. 10.1186/1748-5908-9-55 PMC4019370 24885976

[3] Sherr K, Ásbjörnsdóttir K, Crocker J, Coutinho J, de Fatima Cuembelo M, Tavede E, et al. Scaling-up the Systems Analysis and Improvement Approach for prevention of mother-to-child HIV transmission in Mozambique (SAIA-SCALE): a stepped-wedge cluster randomized trial. Implement Sci. 2019;14(1). Available from: https://implementationscience.biomedcentral.com/articles/10.1186/s13012-019-0889-z. Cited 2020 Feb 20. 10.1186/s13012-019-0889-z PMC6487047 31029171

[4] Wagner AD, Gimbel S, Ásbjörnsdóttir KH, Cherutich P, Coutinho J, Crocker J (2019). Cascade analysis: an adaptable implementation strategy across HIV and non-HIV delivery platforms. JAIDS J Acquir Immune Defic Syndr.

[5] Rustagi AS, Gimbel S, Nduati R, Wasserheit JN, Farquhar C, Gloyd S (2016). Impact of a systems engineering intervention on PMTCT service delivery in Cote d’Ivoire, Kenya, Mozambique: a cluster randomized trial. J Acquir Immune Defic Syndr.

[6] Gimbel S, Mocumbi AO, Ásbjörnsdóttir K, Coutinho J, Andela L, Cebola B, et al. Systems Analysis and Improvement Approach to optimize the hypertension diagnosis and case cascade for PLHIV individuals (SAIA-HTN): a hybrid type III cluster randomized trial. In: Review. 2020. Available from: https://www.researchsquare.com/article/rs-13691/v1. Cited 2021 Mar 24. 10.1186/s13012-020-0973-4 PMC7059349 32143657

[7] Cumbe VFJ, Muanido AG, Turner M, Ramiro I, Sherr K, Weiner BJ, et al. Systems Analysis and Improvement Approach to optimize outpatient mental health treatment cascades in Mozambique (SAIA-MH): study protocol for a cluster randomized trial. Implement Sci. 2022;17(1):37. Available from: https://implementationscience.biomedcentral.com/articles/10.1186/s13012-022-01213-8. Cited 2022 Jul 6. 10.1186/s13012-022-01213-8 PMC9169330 35668423

[8] Fabian KE, Muanido A, Cumbe VF, Manaca N, Hicks L, Weiner BJ (2020). Optimizing treatment cascades for mental healthcare in Mozambique: preliminary effectiveness of the Systems Analysis and Improvement Approach for Mental Health (SAIA-MH). Health Policy Plan.

[9] Beima-Sofie K, Wagner AD, Soi C, Liu W, Tollefson D, Njuguna IN (2022). Providing “a beam of light to see the gaps”: determinants of implementation of the Systems Analysis and Improvement Approach applied to the pediatric and adolescent HIV cascade in Kenya. Implement Sci Commun.

[10] Long JE, Eastment MC, Wanje G, Richardson BA, Mwaringa E, Mohamed MA (2022). Assessing the sustainability of the Systems Analysis and Improvement Approach to increase HIV testing in family planning clinics in Mombasa, Kenya: results of a cluster randomized trial. Implement Sci.

[11] Thomas D, Wanje G, Eastment MC, McClelland RS, Mwaringa E, Patta S (2022). The cost of implementing the Systems Analysis and Improvement Approach for a cluster randomized trial integrating HIV testing into family planning services in Mombasa County, Kenya. BMC Health Serv Res.

[12] Wagner AD, Augusto O, Njuguna IN, Gaitho D, Mburu N, Oluoch G (2022). Systems Analysis and Improvement Approach to optimize the pediatric and adolescent HIV Cascade (SAIA-PEDS): a pilot study. Implement Sci Commun.

[13] Gimbel S, Rustagi AS, Robinson J, Kouyate S, Coutinho J, Nduati R (2016). Evaluation of a Systems Analysis and Improvement Approach to optimize prevention of mother-to-child transmission of HIV using the Consolidated Framework for Implementation Research. JAIDS J Acquir Immune Defic Syndr.

[14] DiCarlo A, Fayorsey R, Syengo M, Chege D, Sirengo M, Reidy W (2018). Lay health worker experiences administering a multi-level combination intervention to improve PMTCT retention. BMC Health Serv Res.

[15] Thomas D, Mujugira A, Ortblad K, Namanda S, Kibuuka J, Nakitende M (2022). A pragmatic approach to identifying implementation barriers and facilitators for a novel pre-exposure prophylaxis (PrEP) delivery model at public facilities in urban Uganda. Implement Sci Commun.

[16] Bardosh KL, Murray M, Khaemba AM, Smillie K, Lester R (2017). Operationalizing mHealth to improve patient care: a qualitative implementation science evaluation of the WelTel texting intervention in Canada and Kenya. Glob Health.

[17] Chayama KL, McNeil R, Shoveller J, Small W, Knight R (2020). Implementation opportunities and challenges identified by key stakeholders in scaling up HIV treatment as prevention in British Columbia, Canada: a qualitative study. Implement Sci Commun.

[18] Roy M, Moore CB, Sikazwe I, Holmes CB (2019). A review of differentiated service delivery for HIV treatment: effectiveness, mechanisms, targeting, and scale. Curr HIV/AIDS Rep.

[19] Gaglio B, Shoup JA, Glasgow RE (2013). The RE-AIM Framework: a systematic review of use over time. Am J Public Health.

[20] Damschroder LJ, Aron DC, Keith RE, Kirsh SR, Alexander JA, Lowery JC. Fostering implementation of health services research findings into practice: a consolidated framework for advancing implementation science. Implement Sci. 2009;4(1). Available from: http://implementationscience.biomedcentral.com/articles/10.1186/1748-5908-4-50. Cited 2019 May 24. 10.1186/1748-5908-4-50 PMC2736161 19664226

[21] Kirk MA, Kelley C, Yankey N, Birken SA, Abadie B, Damschroder L. A systematic review of the use of the Consolidated Framework for Implementation Research. Implement Sci. 2015;11(1). Available from: http://implementationscience.biomedcentral.com/articles/10.1186/s13012-016-0437-z. Cited 2020 Jan 21. 10.1186/s13012-016-0437-z PMC4869309 27189233

[22] Means AR, Kemp CG, Gwayi-Chore MC, Gimbel S, Soi C, Sherr K, et al. Evaluating and optimizing the Consolidated Framework for Implementation Research (CFIR) for use in low- and middle-income countries: a systematic review. Implement Sci. 2020;15(1). Available from: https://implementationscience.biomedcentral.com/articles/10.1186/s13012-020-0977-0. Cited 2020 Oct 21. 10.1186/s13012-020-0977-0 PMC7069199 32164692

[23] Soi C, Gimbel S, Chilundo B, Muchanga V, Matsinhe L, Sherr K. Human papillomavirus vaccine delivery in Mozambique: identification of implementation performance drivers using the Consolidated Framework for Implementation Research (CFIR). Implement Sci. 2018;13(1). Available from: https://implementationscience.biomedcentral.com/articles/10.1186/s13012-018-0846-2. Cited 2019 May 24. 10.1186/s13012-018-0846-2 PMC6293623 30545391

[24] Cole CB, Pacca J, Mehl A, Tomasulo A, van der Veken L, Viola A, et al. Toward communities as systems: a sequential mixed methods study to understand factors enabling implementation of a skilled birth attendance intervention in Nampula Province, Mozambique. Reprod Health. 2018;15(1). Available from: https://reproductive-health-journal.biomedcentral.com/articles/10.1186/s12978-018-0574-8. Cited 2020 Oct 22. 10.1186/s12978-018-0574-8 PMC6091088 30075791

[25] The AHI PHIT Partnership Collaborative, Gimbel S, Mwanza M, Nisingizwe MP, Michel C, Hirschhorn L. Improving data quality across 3 sub-Saharan African countries using the Consolidated Framework for Implementation Research (CFIR): results from the African Health Initiative. BMC Health Serv Res. 2017;17(S3). Available from: https://bmchealthservres.biomedcentral.com/articles/10.1186/s12913-017-2660-y. Cited 2020 Jan 21. 10.1186/s12913-017-2660-y PMC5763292 29297401

[26] The AHI PHIT Partnership Collaborative, Rwabukwisi FC, Bawah AA, Gimbel S, Phillips JF, Mutale W, et al. Health system strengthening: a qualitative evaluation of implementation experience and lessons learned across five African countries. BMC Health Serv Res. 2017;17(S3). Available from: https://bmchealthservres.biomedcentral.com/articles/10.1186/s12913-017-2662-9. Cited 2020 Oct 22. 10.1186/s12913-017-2662-9 PMC5763488 29297333

[27] Burawoy M (1998). The extended case method. Sociol Theory.

[28] Burawoy M (2009). The extended case method: four countries, four decades, four great transformations, and one theoretical tradition.

[29] Gluckman M (1940). Analysis of a social situation in modern Zululand. Bantu Stud.

[30] Mol A. The logic of care. Health Probl Patient Choice. 2008. Available from: http://samples.sainsburysebooks.co.uk/9781134053179_sample_509843.pdf. Cited 2015 Feb 2.

[31] Miles MB, Huberman MA, Saldana J (2014). Qualitative data analysis: a methods sourcebook.

[32] Hennink MM, Kaiser BN, Marconi VC (2017). Code saturation versus meaning saturation: how many interviews are enough?. Qual Health Res.

[33] Baker SE, Edwards R (2012). How many qualitative interviews is enough? Expert voices and early career reflections on sampling and cases in qualitative research.

[34] Guest G (2006). How many interviews are enough?: an experiment with data saturation and variability. Field Methods.

[35] Schoepfle GM, Werner O (1999). Ethnographic debriefing. Field Methods.

[36] Inguane C, Soi C, Gimbel S, Manaca N, Ramiro I, Floriano F (2022). Applying the Consolidated Framework for Implementation Research to identify implementation determinants for the integrated district evidence-to-action program, Mozambique. Glob Health.

[37] Damschroder LJ, Lowery JC (2013). Evaluation of a large-scale weight management program using the consolidated framework for implementation research. Implement Sci..

[38] Tong A, Sainsbury P, Craig J (2007). Consolidated Criteria for Reporting Qualitative Research (COREQ): a 32-item checklist for interviews and focus groups. Int J Qual Health Care.

[39] VanDevanter N, Kumar P, Nguyen N, Nguyen L, Nguyen T, Stillman F, et al. Application of the Consolidated Framework for Implementation Research to assess factors that may influence implementation of tobacco use treatment guidelines in the Viet Nam public health care delivery system. Implement Sci. 2017;12(1). Available from: https://implementationscience.biomedcentral.com/articles/10.1186/s13012-017-0558-z. Cited 2020 Nov 24. 10.1186/s13012-017-0558-z PMC5330005 28241770

[40] Rustagi AS, Gimbel S, Nduati R, Cuembelo MDF, Wasserheit JN, Farquhar C (2017). Health facility factors and quality of services to prevent mother-to-child HIV transmission in Côte d’Ivoire, Kenya, and Mozambique. Int J STD AIDS.

